# Effects and central mechanisms of acupuncture for post-stroke vascular vertigo: study protocol of a multicenter, randomized, sham-controlled trial

**DOI:** 10.3389/fneur.2026.1729679

**Published:** 2026-03-25

**Authors:** Lina Pang, Shufang Li, Meng Gong, Xiangyin Ye, Chunyan Gou, Xiang Mao, Renyan Xiao, Yan Li, Xinyi Li, Hongyuan Wang, Song Jin

**Affiliations:** 1 College of Acupuncture and Tuina, Chengdu University of Traditional Chinese Medicine, Chengdu, China; 2 College of Health Preservation and Rehabilitation, Chengdu University of Traditional Chinese Medicine, Chengdu, China; 3 Chongqing Traditional Chinese Medicine Hospital, Chongqing, China; 4 College of Medical and Life Sciences, Chengdu University of Traditional Chinese Medicine, Chengdu, China; 5 Hospital of Chengdu University of Traditional Chinese Medicine, Chengdu, China

**Keywords:** acupuncture, central mechanisms, randomized controlled trial, stroke, vascular vertigo

## Abstract

**Background:**

Vascular vertigo is a common sequela following stroke, significantly impairing patients’ quality of life and rehabilitation progress. Although Western pharmaceutical treatments are widely used, their long-term efficacy is limited and associated with side effects. Acupuncture, a traditional Chinese medical therapy, has potential advantages in alleviating vertigo symptoms. This study aims to evaluate the efficacy and central mechanisms of acupuncture for post-stroke vascular vertigo (PSVV).

**Methods:**

This study is a multicenter, double-blind, randomized controlled trial planned to enroll 234 patients with PSVV from four subcenters. Participants will be randomly assigned in a 1:1:1 ratio to the acupuncture group, the sham acupuncture group, or the active drug group. The trial comprises a 1-week induction period, a 3-week treatment period, and follow-up periods at 8 weeks and 20 weeks. Following the induction period, the drug group will receive betahistine mesilate tablets orally, the acupuncture group will receive needling at Baihui (GV20), Fengchi (GB20), Wangu (GB12), and Taichong (LR3), and the sham acupuncture group will receive superficial needling at non-acupoints combined with blunt needle. All groups will undergo intervention 3 weeks. The primary outcome will be the Dizziness Handicap Inventory (DHI), with secondary outcomes including the Dizziness and Anxiety Rating Scale (DARS), dizziness diaries, the 36-Item Short Form Health Survey (SF-36), transcranial Doppler ultrasonography (TCD), multimodal magnetic resonance imaging (MRI), and Expectation Rating Scale (ERS). The relationship between clinical efficacy and the mechanisms of the prescribed interventions will be explored. Treatment safety will be assessed through adverse event records.

**Discussion:**

This trial will be the first to compare the efficacy of acupuncture, sham acupuncture, and Western medication in treating PSVV. It aims to demonstrate whether acupuncture is safe and effective for this condition and to elucidate its central nervous system mechanisms. We anticipate that this research will provide clinical evidence for the management of PSVV.

**Ethics and dissemination:**

The Medical Ethics Committee of the Chengdu University of Traditional Chinese Medicine Affiliated Hospital has given study approval (approval no. 2025KL-062). The findings will be disseminated through publications, conferences, and briefs to professional organizations.

**Clinical trial registration:**

http://itmctr.ccebtcm.org.cn, identifier ITMCTR2025001283.

## 1 Introduction

Recently, diagnostic criteria for Vascular Vertigo were presented by the Bárány Society’s Committee for the Classification of Vestibular Disorders. Vascular vertigo refers to vertigo caused by vascular lesions, typically presenting as acute vestibular syndrome (AVS), characterized by the acute onset of vertigo with nausea or vomiting, head-motion intolerance, and unsteadiness (1). The classification includes vertigo due to stroke or transient ischemic attack as well as vertebral artery compression syndrome, and isolated labyrinthine infarction/hemorrhage, with stroke being the common cause of vascular vertigo (2). Vertigo is also an independent risk factor for stroke patients (3, 4). Post-stroke vascular vertigo (PSVV) is primarily caused by cerebral ischemia or hemorrhage, encompassing both anterior circulation and posterior circulation strokes (5, 6). Post-stroke recovery of cognition, aphasia, upper limb, mobility, and fatigue has received particular attention, yet the accompanying symptom of vertigo is often overlooked (7, 8). Furthermore, recurrent episodes of vertigo increase the risk of stroke recurrence and mortality (9).

The rehabilitative effect of acupuncture, the most widely used complementary alternative medicine method for stroke, has gained international recognition (10). In recent years, acupuncture treatment for vascular vertigo has gained increasing attention among clinicians. Clinical studies indicate that acupuncture is safe and effective for patients experiencing vertigo following a stroke (11). However, the advantages and the underlying mechanisms of acupuncture and moxibustion in the treatment of PSVV have not yet been fully elucidated.

Multimodal magnetic resonance imaging (MRI) is widely used in clinical practice and research of neurological and psychiatric disorders, as it visually reflects brain function and structural changes—making it a key technique in stroke research (12). Specifically, resting-state functional MRI (rs-fMRI), a connectomics tool, focuses on short-term brain network connectivity changes (13) and aids in vertigo prognosis assessment (14). Research has found that vertigo involves multiple vestibular and non-specific networks, and is associated with the cortico-basal ganglia-cerebellum-thalamus network (15). Meanwhile, white matter plays a crucial role in vertigo and imbalance caused by vestibular dysfunction (16). Diffusion tensor imaging (DTI) quantifies the integrity, directionality, and connectivity of white matter fiber tracts by detecting the diffusion characteristics of water molecules within brain tissue (17). Additionally, DTI can investigate differences in connectivity within the vestibular projection pathways (18). Therefore, exploring the neuroplasticity underlying acupuncture treatment for vascular vertigo using these two neuroimaging techniques holds both fundamental and clinical significance.

Therefore, this multicenter randomized controlled trial will evaluate the efficacy of acupuncture in treating PSVV and explore the central mechanisms underlying acupuncture’s therapeutic effects in alleviating vertigo symptoms among stroke patients.

## 2 Study design and methods

### 2.1 Objectives

This study has two primary objectives: (1) compare the clinical efficacy of real acupuncture, sham acupuncture, and active drugs for PSVV to verify acupuncture’s efficacy and safety for this condition; (2) explore the central nervous system mechanisms of acupuncture for PSVV using multimodal MRI.

### 2.2 Design and setting

**Figure 1:**
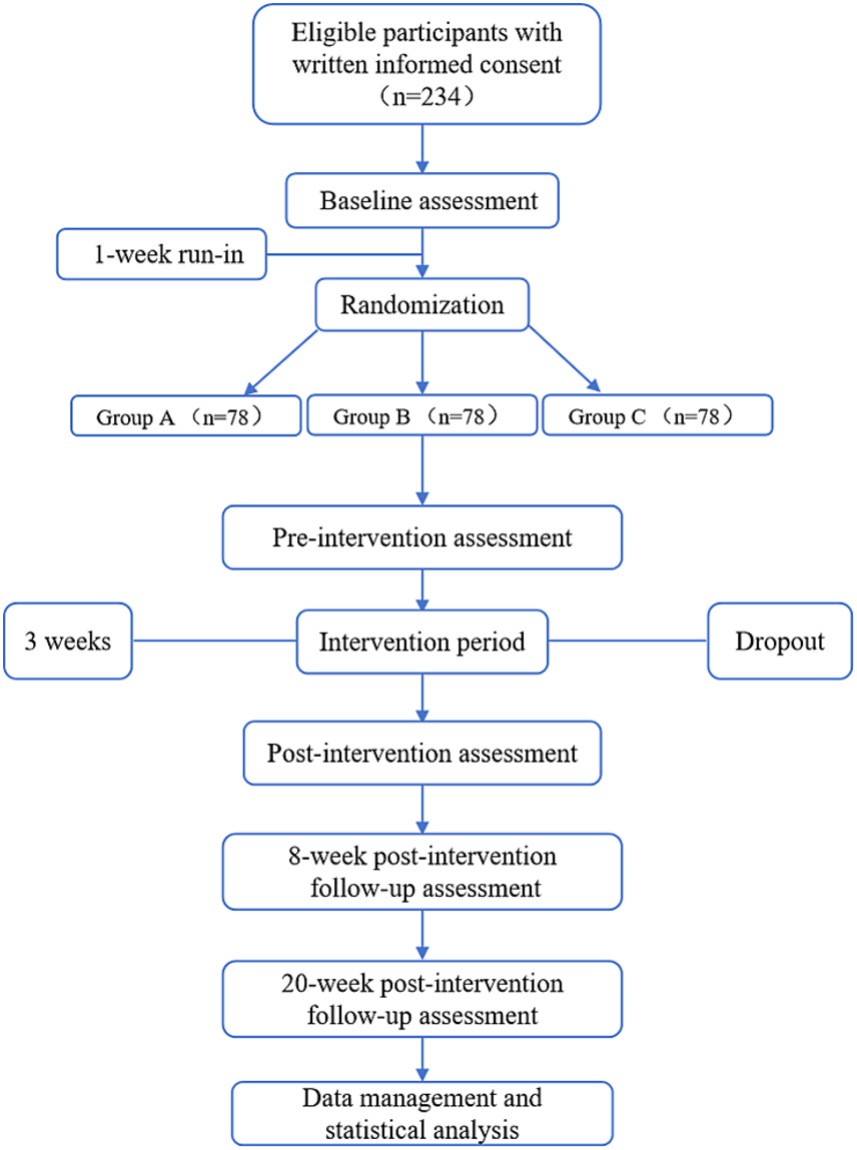
Flowchart of the research procedure.

This is a multicenter, double-blind, randomized, controlled study that meets the recommendations of the Standard Protocol Project: Intervention Trials guidelines (19). This trial will be conducted in four clinical subcenters: the Affiliated Hospital of Chengdu University of Traditional Chinese Medicine, Chongqing Hospital of Traditional Chinese Medicine, Shuangliu District Hospital of Traditional Chinese Medicine in Chengdu, and Longquanyi District Hospital of Traditional Chinese Medicine in Chengdu. A total of 234 eligible participants will be randomly assigned to the active drug group, the acupuncture group, and the sham acupuncture group (1:1:1 allocation ratio). Participants will be evaluated at baseline, week 3, week 11, and week 23. Transcranial Doppler ultrasonography (TCD) and multimodal MRI will be performed at baseline and at week 3. All outcome assessors will be blinded to group assignment. The flowchart and results evaluation schedule of this trial are shown in Figure 1 and Table 1. The protocol was approved by the Medical Ethics Committee of the Affiliated Hospital of Chengdu University of Traditional Chinese Medicine (approval no. 2025KL-062) (Supplementary File 1) and is registered with the International Traditional Medicine Clinical Trial Registration Platform (registration no. ITMCTR2025001283).

**Table 1:** Study schedule for data measurements.

Timepoint	Study period				
	Enrolment	Allocation	Treatment	Follow-up	
	Week-1	Week 0	Week 3	Week 11	Week 23
Enrolment					
Eligibility screen	×				
Informed consent	×				
Physical examination	×				
PMH and PTH	×				
CD or CM	×				
Randomization		×			
Interventions					
Group A (*n* = 78)		×	×		
Group B (*n* = 78)		×	×		
Group C (*n* = 78)		×	×		
Assessments					
DHI		×	×	×	×
DARS		×	×	×	×
Vertigo diary		×	×	×	×
SF-36		×	×	×	×
TCD		×	×		
fMRI parameters		×	×		
ERS		×			
Participants safety					
AE		×	×	×	×

*Group A, acupuncture group; Group B, sham acupuncture group; Group C, active drug group; PMH, past medical history; PTH, prior therapy history; CD, concomitant diseases; CM, concomitant medications; Group A, acupuncture group; Group B, sham acupuncture group; Group C, drug control group; DHI, Dizziness Handicap Inventory; DARS, Dizziness and Anxiety Rating Scale; SF-36, 36-Item Short Form Health Survey; TCD, transcranial Doppler ultrasonography; fMRI, functional magnetic resonance imaging; ERS, Expectation Rating Scale; AE, Adverse Event.

### 2.3 Participants

#### 2.3.1 Recruitment and informed consent

Participants will be recruited from the outpatient and inpatient departments of the Affiliated Hospital of Chengdu University of Traditional Chinese Medicine, Chongqing Hospital of Traditional Chinese Medicine, Chengdu Shuangliu District Hospital of Traditional Chinese Medicine, and Chengdu Longquanyi Hospital of Traditional Chinese Medicine. Recruitment is scheduled to commence on July 1, 2025. Investigators will strictly adhere to the inclusion and exclusion criteria to determine each individual’s eligibility for the study.

All potential participants will be informed of the study’s purpose and nature, and provided with comprehensive information about the research, including its procedures, potential benefits, and risks. Before enrollment, they will voluntarily sign an informed consent form (Supplementary File 2). Participants reserve the right to withdraw from the study at any time without penalty or loss of benefits, and are not required to provide a reason for withdrawal. Any reasons for withdrawal, if provided, will be documented.

#### 2.3.2 Inclusion criteria

According to the Bárány Society (2), vascular vertigo can result from either central lesions (brainstem, cerebellar, or supratentorial) or isolated labyrinthine infarction/hemorrhage. In the present study, only patients with central vascular vertigo following ischemic or hemorrhagic stroke are eligible.

Inclusion requires: (1) neuroimaging evidence of a lesion involving the vertebrobasilar territory or supratentorial central vestibular structures (e.g., brainstem, cerebellum, thalamus, insula, temporo-parietal cortex) that is judged by the investigator to be responsible for the vertigo; (2) aged 30–80 years (inclusive), regardless of gender; (3) ≥ 2 weeks since the onset of cerebral infarction or cerebral hemorrhage, with persistent vertigo or postural instability for ≥1 week (vertigo-related symptoms must not precede the diagnosis of cerebral infarction/hemorrhage); (4) Barthel Index (BI) score >40; (5) stable vital signs and overall clinical condition; ability to complete a vertigo diary as instructed; (6) not participating in any other trials during the study period and signing a written informed consent form.

#### 2.3.3 Exclusion criteria

Participants will be excluded from this study if they meet any of the following criteria: (1) other primary peripheral vestibular disorders (e.g., Ménière disease, vestibular neuritis, benign paroxysmal positional vertigo), including isolated vascular events affecting solely the peripheral vestibular apparatus (e.g., labyrinthine infarction/hemorrhage) with no concomitant central nervous system involvement; (2) they have vascular vertigo associated with vertebral artery compression syndrome; (3) vertigo caused by non-vascular factors (e.g., intracranial space-occupying lesions, otogenic diseases, metabolic/infectious diseases, trauma) confirmed by examination; (4) they have comorbid severe primary diseases involving the cardiovascular system, liver, kidneys, digestive system, hematopoietic system, or other major organ systems; (5) they have been diagnosed with other central nervous system diseases within the past 6 months; (6) there are severe skin lesions or skin diseases at the treatment site; (7) they have coagulation disorders or hemorrhagic diseases; (8) pregnant, lactating, or females planning pregnancy within 6 months; (9) they are unable to understand the content of the vertigo diary or complete the required recordings; (10) they are in a comatose state or have consciousness disorders; (11) they are unable to cooperate with all study procedures due to mental illness, cognitive impairment, or emotional disorders; (12) current participation in another clinical trial or participation in one within the past 3 months.

### 2.4 Baseline characterization and lesion documentation

For all enrolled patients, the location of the responsible lesion will be documented based on the qualifying MRI or CT scan. Lesion location will be classified *a priori* into three categories: (1) brainstem lesions; (2) cerebellar lesions; and (3) supratentorial central vestibular lesions (e.g., thalamic, temporo-parietal, insular). In cases with combined lesions, the predominant clinically relevant site will be recorded. The distribution of patients across these lesion categories will be summarized in the baseline characteristics table. This categorization will also serve as a basis for exploratory subgroup analyses to investigate potential differential treatment responses based on neuroanatomical substrate.

### 2.5 Sample size

The sample size was calculated on the basis of the primary outcome, change in total DHI score. The assumed mean DHI scores were derived from a previous randomized trial on vestibular vertigo (20) and supported by a pilot study of 30 PSVV patients conducted by our team prior to this trial. The pilot data indicated a mean DHI reduction of approximately 12 points in the acupuncture group compared to sham, which informed our assumed group differences. On thess evidence, this study assumes that the mean Dizziness Handicap Inventory (DHI) score will be 21 in the acupuncture group for post-stroke vascular vertigo, 33 in the sham acupuncture group, and 30 in the active drug group, with a predefined standard deviation (SD) of 20. Sample size calculation will be performed using PASS software, with a set power (1-*β*) of 0.90 and a significance level (*α*) of 0.05. Following this calculation, a minimum of 66 participants will be required for each group, yielding a total sample size of 198; considering a 15% attrition rate, the total sample size will be adjusted to 234, with no fewer than 78 participants in each group.

### 2.6 Randomization and blinding

All eligible participants will be randomly assigned to three groups at a 1:1:1 ratio using computer-generated random numbers sealed in opaque envelopes. Dedicated personnel responsible for randomization will open the envelopes in enrollment order and inform treating physicians of group assignments. All steps in the implementation of this trial’s randomization protocol will be conducted by dedicated randomization staff who are not involved in the trial itself. Blinding of the acupuncture practitioners will not be feasible due to the nature of the interventions. Nevertheless, the principle of blinding will be consistently upheld throughout the trial: participants in the acupuncture group and sham acupuncture group will receive treatment in separate, partitioned rooms to prevent interaction with other groups. After the 3-week treatment period, a blinding assessment will be conducted for these two groups to evaluate the perceived treatment type (real acupuncture, sham acupuncture, or undetermined) based on participants’ experience. Throughout the trial, the principle of separating researchers, operators, and statisticians will be strictly followed.

### 2.7 Interventions

All participants will receive basic treatment prior to intervention. Per the *Chinese Guidelines for Secondary Prevention of Ischemic Stroke and Transient Ischemic Attack* (21), this basic treatment includes three aspects: (1) Controlling risk factors such as blood pressure and blood glucose; (2) Administering regular-dose aspirin for antiplatelet aggregation (if no contraindications) and atorvastatin for lipid regulation and plaque stabilization; (3) Providing health education on diet, lifestyle, and physical activity. Participants in the acupuncture and sham acupuncture groups will receive a 3-week intervention: one 30-min session daily, 5 times weekly (2 rest days on weekends). The active drug group will take betahistine mesilate tablets orally three times daily for three consecutive weeks.

**Figure 2:**
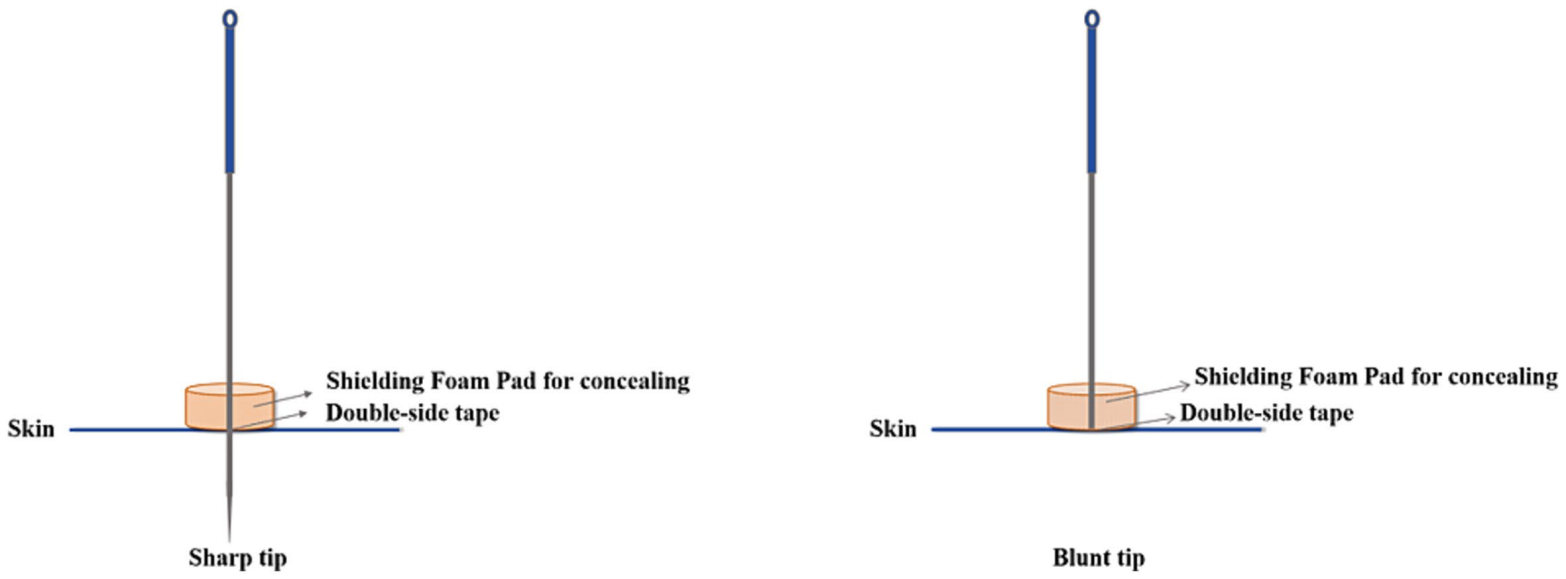
Streitberger sham acupuncture device.

**Figure 3:**
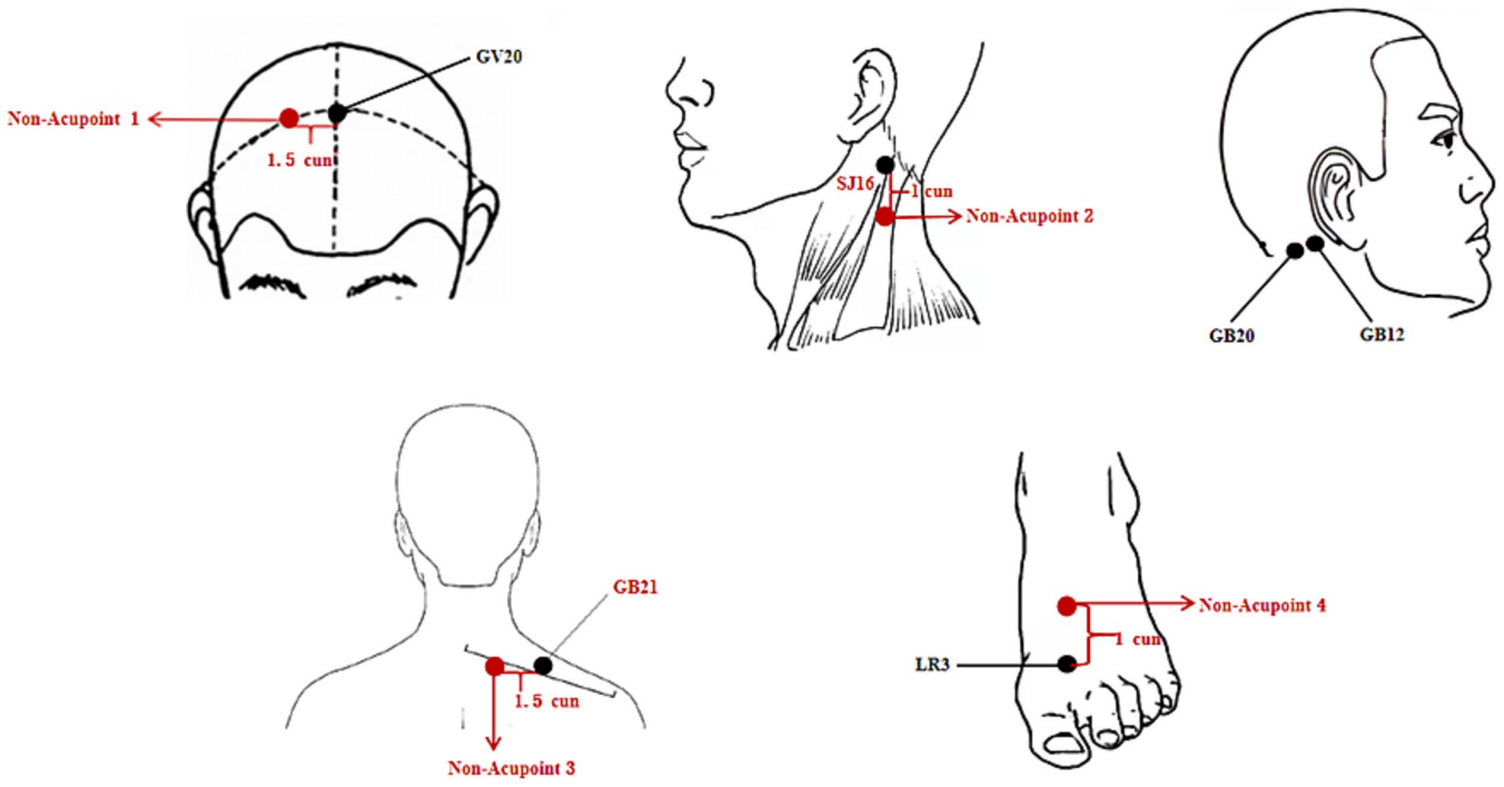
Location of acupoints and non-acupoints.

In the acupuncture group (Group A), participants will undergo acupuncture with filiform needles (Huatuo brand, Suzhou, China; Figure 2) at the following acupoints: Baihui (GV20), bilateral Fengchi (GB20), bilateral Wangu (GB12), and bilateral Taichong (LR3) (Figure 3; Table 2). After needling the acupoints, needles will be twisted 90–180° and lifted and inserted by 3–5 mm to induce a *deqi* sensation. This stimulation will be applied immediately after insertion and then repeated at 10-min intervals during the 30-min needle retention period, yielding three stimulation episodes per treatment session.

**Table 2:** Location of acupoints and non-acupoints.

Acupoints and non-acupoints	Location
GV20 (Baihui)	On the head vertex, at the midpoint of the line connecting the two ear apices, 5 cun above the anterior hairline midpoint.
GB20 (Fengchi)	In the anterior region of the neck, inferior to the occipital bone, in the depression between the origins of sternocleidomastoid and the trapezius muscles.
GB12 (Wangu)	On the lateral head (posterior to the ear), in the depression inferior to the mastoid process at the sternocleidomastoid attachment.
LR3 (Taichong)	On the foot dorsum, 1 cun proximal to the 1st-2nd metatarsophalangeal joint, between the 1st and 2nd metatarsal bones.
Non-acupoint 1	1.5 cun lateral to GV20 (Baihui)
Non-acupoint 2	1 cun inferior to GB9 (Tianyou, on the lateral neck, posterior to the sternocleidomastoid muscle, at thyroid cartilage upper border level).
Non-acupoint 3	1.5 cun lateral to GB21 (Jianjing, on upper back, midpoint of C7 spinous process-acromion line, approximately 4 cun lateral to posterior midline).
Non-acupoint 4	1 cun superior to LR3 (Taichong).

In the sham acupuncture group (Group B), participants will receive treatment with retractable blunt needles (Streitberger type, Suzhou, China; Figure 2) at non-acupoints (Figure 3; Table 2), using skin-non-penetrating stimulation.

In the active drug group (Group C), participants will receive betahistine mesilate tablets (trade name: Minshilang).

### 2.8 Outcome measures

#### 2.8.1 Primary outcome

The primary outcome will be assessed using the DHI. The DHI yields a total score of 0–100, with three subscales: Physical (P, 28 points), Emotional (E, 36 points), and Functional (F, 36 points). A higher total score is indicative of greater functional impairment attributable to dizziness, whereas a lower score denotes less severe dizziness-related disability (Supplementary File 3).

#### 2.8.2 Secondary outcomes

##### 2.8.2.1 Dizziness and Anxiety Rating Scale (DARS)

This scale quantifies vertigo severity by rating standing balance dysfunction, gait balance impairment, current vertigo status, confusion or disorientation, and global impression of disease severity (as rated by both physicians and patients). Total scores range from 0 to 36, with higher scores indicating more severe vertigo (Supplementary File 4).

##### 2.8.2.2 Vertigo diary

Participants will be instructed to record comprehensive details of each vertiginous episode in a vertigo diary, including episode frequency, onset and offset timestamps, vertigo Visual Analogue Scale (VAS) scores, and severity gradings. The diary will be completed manually by participants upon cessation of each episode and subject to regular monitoring by dedicated research personnel (Supplementary File 5).

##### 2.8.2.3 36-Item Short Form Health Survey (SF-36)

This scale includes domains of physical functioning, role-physical, bodily pain, role-emotional, mental health, vitality, social functioning, and general health. Higher scores indicate better quality of life (Supplementary File 6).

##### 2.8.2.4 TCD

TCD will be used to monitor cerebral blood flow (CBF), as it is well-established for non-invasive intracranial hemodynamic assessment. Two clinically validated indices of CBF status will be measured: Mean Flow Velocity (MFV) and Resistance Index (RI). A standardized protocol will be applied to 9 target intracranial vessels, specifically the left middle cerebral artery (L-MCA), right middle cerebral artery (R-MCA), left anterior cerebral artery (L-ACA), right anterior cerebral artery (R-ACA), left posterior cerebral artery (L-PCA), right posterior cerebral artery (R-PCA), basilar artery (BA), left vertebral artery (L-VA), and right vertebral artery (R-VA).

##### 2.8.2.5 fMRI data acquisition and scanning parameters

All fMRI data will be acquired using a 3.0 T GE Discovery 750 MRI scanner (Milwaukee, WI, USA) at the Affiliated Hospital of Chengdu University of Traditional Chinese Medicine. During scanning, participants’ heads will be immobilized with customized foam pads, and dedicated earplugs will be provided to minimize head motion artifacts and reduce noise.

Data acquisition will include axial structural imaging using a T1-weighted fast spoiled gradient echo (FSPGR) sequence, positioned perpendicular to the anterior commissure-posterior commissure (AC-PC) line, covering the entire brain from the vertex to the skull base. Parameters: repetition time (TR) = 7 ms, echo time (TE) = 3.1 ms, inversion time (TI) = 450 ms, field of view (FOV) = 240 mm × 240 mm, matrix = 256 × 256, slice thickness = 1.0 mm, flip angle = 15°, and scan time = 246 s. Resting-state fMRI (rs-fMRI) will be acquired using a single-shot gradient echo-planar imaging (GRE-EPI) sequence. Parameters: repetition time (TR) = 2000 ms, echo time (TE) = 30 ms, field of view (FOV) = 240 × 240 mm^2^, matrix = 64 × 64, slice thickness = 4.0 mm, flip angle = 90°, and scan time = 300 s. Diffusion Tensor Imaging (DTI) will be acquired using a whole-brain diffusion-weighted single-shot spin-echo EPI sequence. Parameters: repetition time (TR) = 6,800 ms, echo time (TE) = 86.3 ms, field of view (FOV) = 256 × 256 mm^2^, matrix = 120 × 120, slice thickness = 3.0 mm, flip angle = 90°, scan time = 224 s.

##### 2.8.2.6 Expectation Rating Scale (ERS)

Participants in each group were asked, “What is your expected efficacy of the treatment for vascular vertigo?” with the following response options: “Excellent efficacy” (symptom improvement >75%); “Moderate efficacy” (symptom improvement 50–75%); “Slight efficacy” (symptom improvement 25–50%); and “No change” (symptom improvement 0–25%). “Excellent efficacy” was defined as high expectation, while the remaining options were defined as low expectation. This assessment was conducted at the time of at baseline. To minimize confounding factors, the same physician, who was blinded to the specific treatment allocation, performed the evaluation for all participants (Supplementary File 7).

#### 2.8.3 Safety outcomes

Adverse events (AEs) include acupuncture-related events (bleeding, hematoma, pain, infection, syncope) and betahistine mesilate-related adverse reactions (gastrointestinal symptoms, rashes, other allergic responses). All AEs will be documented in detail on the Case Report Form (CRF) when they occur. For serious adverse events (SAEs—defined as death, life-threatening conditions, disability, or hospitalization), the principal investigator must report to the trial site and hospital ethics committees within 24 h, with accurate CRF documentation. All AEs will be followed up until resolution, and the ethics committee will determine whether to terminate the trial if necessary.

### 2.9 Quality control

To ensure trial quality, the principal investigator will provide training to all study staff on operational procedures, outcome measures, and eligibility criteria. The principal investigator will also oversee the study to maintain data accuracy. Case Report Forms (CRFs) and study data will be securely stored, with key documents retained for 5 years post-publication. Any protocol deviations will be reported to the ethics committee, which will determine whether to modify or terminate the study.

### 2.10 Statistical methods

#### 2.10.1 Clinical data analysis

Clinical outcomes will be analyzed using SPSS 23.0 statistical software, with a significance level of *p*-value < 0.05. All analyses will be performed by independent statisticians blinded to group assignments and interventions. Two core analysis sets will be used: (1) Full Analysis Set (FAS): All randomly assigned participants who received at least one treatment session. Missing values for primary outcomes will be imputed using the Last Observation Carried Forward (LOCF) method, and FAS results will be the primary analytical basis. (2) Per Protocol Set (PPS): Participants with no exclusion criterion violations, ≥10 completed acupuncture sessions, and at least the Week 3 assessment. PPS results will serve as supplementary references. Relevant statistical analyses will also cover case distribution analysis, which will calculate the sample size of each analysis set per group, case distribution across study centers, and total numbers of dropouts and withdrawals (with respective reasons), and present these data in detailed tables.

The correlation between baseline treatment expectation and changes in DHI will be examined using Pearson correlation. In addition, multiple linear regression models will be constructed with change in DHI as the dependent variable, and baseline expectation score, treatment group, baseline DHI, age, and sex as independent variables, to examine whether expectation independently predicts treatment response. Exploratory interaction terms between treatment group and expectation will be tested to evaluate whether the magnitude of the acupuncture effect differs according to baseline expectation level.

#### 2.10.2 fMRI data analysis

##### 2.10.2.1 T1-weighted data

Whole-brain gray matter volume (GMV) will be calculated from 3D T1-weighted images using voxel-based morphometry (VBM) in FSL 5.0.9.1 software. Workflow: ① Remove non-brain tissues using the Brain Extraction Tool (BET). ②Segment brain tissues into gray matter, white matter, and cerebrospinal fluid using the FMRIB Automated Segmentation Tool (FAST). ③Nonlinearly register individual gray matter images to the ICBM-152 standard gray matter template using the FNIRT tool, then construct a preliminary study-specific template via averaging and x-axis flipping. ④Re-register individual gray matter images to the preliminary template, then average to generate a final symmetric study-specific template. ⑤Modulate registered gray matter images using the Jacobian matrix of the deformation field to produce GMV maps. ⑥ Smooth GMV images with a 3 mm Gaussian kernel (full width at half maximum, FWHM ≈ 7 mm).

##### 2.10.2.2 rs-fMRI data

① *Functional Connectivity (FC)*: Defined as the temporal correlation of neurophysiological indices across brain regions, depicting the relationship between neuronal activity patterns of anatomically separated regions and reflecting functional communication levels.

② *Amplitude of Low-Frequency Fluctuations (ALFF)*: Quantifies the intensity of spontaneous low-frequency neural oscillations, directly reflecting local brain functional status.

③ *Regional Homogeneity (ReHo)*: Reflects local brain function by quantifying the synchrony of blood-oxygen-level-dependent (BOLD) time series between a voxel and its neighbors using Kendall’s coefficient of concordance. Synchrony variations are closely associated with neural activity and brain function changes (22).

④ *BOLD*: fMRI uses BOLD signals to dynamically represent brain activity. In the resting state, spontaneous BOLD fluctuations show spatial synchrony across functionally relevant brain regions—enabling identification of multiple resting-state functional connectivity networks. Abnormalities and recovery of functional connectivity in networks such as the default mode network (DMN) and executive control network (ECN) are directly linked to post-stroke recovery. These abnormalities correlate with post-stroke white matter structural damage, illuminating the relationship between structural injury and functional deficits. We aim to investigate DMN-related functional connectivity by examining BOLD signal changes across brain regions (23).

##### 2.10.2.3 DTI data

① *Fractional Anisotropy (FA)*: A quantitative index of anisotropic diffusion in DTI, enabling detection of subtle changes in white matter diffusion properties. FA values reflect axonal integrity, with abnormalities indicating axonal damage. Baseline whole-brain mean FA is associated with subsequent vertigo severity changes (24). We will extract pre- and post-treatment mean FA values of the whole-brain white matter skeleton for each patient and correlate them with DHI score changes to explore whether acupuncture ameliorates vertigo by modulating white matter integrity.

② *Mean Diffusivity (MD)*: Quantifies overall water molecule diffusion freedom, indirectly reflecting physiological structural integrity and pathophysiological changes in the nervous system. Elevated MD indicates impaired fiber tract integrity, leading to delayed/disrupted neural signal transmission and functional connectivity abnormalities (25).

Furthermore, the Pearson correlation analysis between the clinical results and fMRI results will be conducted to investigate the potential correlation between symptom improvements and brain activity changes elicited by different interventions.

## 3 Discussion

The Bárány Society published diagnostic criteria for vascular vertigo in 2022 after a decade of research (2). It should be emphasized that, although the Bárány Society’s definition of vascular vertigo includes both central and peripheral vascular lesions, the present trial is restricted to PSVV. As a result, the findings of this study will be directly applicable to patients with brainstem, cerebellar, or other central vestibular stroke sequelae, but not to isolated labyrinthine infarction or other purely peripheral vascular vestibular syndromes, which require separate investigation. Based on these criteria, this study designs a multicenter, randomized, double-blind trial to evaluate acupuncture’s efficacy for PSVV and explore neuroplasticity mechanisms. This trial is the first to examine a standardized acupuncture protocol for PSVV and conduct neuroimaging research. Combining DTI and rs-fMRI results will enable evaluation of white matter microstructure and gray matter function improvements after acupuncture.

Notably, sensations such as dizziness, vertigo, and unsteadiness can be subjective and poorly defined in stroke patients’ self-reports, so clinicians must discern them more clearly (26). Vertigo is a motion illusion from spatial disorientation, with patients perceiving themselves/surroundings as spinning, tumbling, or tilting. Dizziness is described as head heaviness, lightheadedness, or floating. Unsteadiness presents as unstable gait, imbalance, or a feeling of impending fall—typically absent when lying/sitting. A study reported a significantly increased prevalence of balance problems and associated psychiatric symptom burden (27). PSVV primarily arises from central vestibular pathway damage after stroke (28). Some patients may develop Post-stroke pusher syndrome, primarily due to impairments in proprioception, cerebellar coordination, and damage to pyramidal or extrapyramidal systems. It is characterized by active tilting toward the hemiplegic side and resistance to correction (29). These conditions are distinct from true PSVV and require careful differentiation.

Acupuncture is a multifactorial comprehensive therapy, making control group design complex. Sham acupuncture is one method to evaluate acupuncture’s specific therapeutic effects (30, 31). Previous studies used acupuncture as adjunctive therapy, with small sample sizes and poorly designed control groups (32). These limitations have prevented a clear determination of acupuncture’s intrinsic role in treating PSVV. Thus, this study increases sample size, expands population scope, and uses the Streitberger device as sham control to evaluate acupuncture’s specific effects on PSVV. Concurrently, patient treatment expectations will be quantified, and their association with clinical efficacy will be explored through correlation analysis and multiple regression analysis. Additionally, the conclusions will explore the relationship between white matter microstructure, gray matter function, and vertigo improvement based on rs-fMRI, DTI, and vertigo assessment scale results, aiming to provide further evidence for the mechanism of acupuncture’s efficacy in treating PSVV.

To ensure reliable results, participants will be randomly assigned to groups, with outcome assessors and statisticians blinded to group assignments to minimize potential bias. However, this trial has several limitations. Owing to the inability to blind among the acupuncturists, the use of self-reported outcomes, and the potential placebo effect of acupuncture, the study results may be prone to bias. Additionally, a challenge for clinical applications of rs-fMRI is the potentially confounding effect of concomitant vascular diseases, aging, or medication on the neurovascular coupling and consequently the fMRI imaging response (33). Therefore, quality control methods are prerequisites for reliable results: (1) recruit right-handed patients for MRI to avoid dominant hand effects on brain structure and function; (2) acupuncture procedures will be strictly performed by trained, certified practitioners with at least 3 years of experience following standardized protocols; (3) Standardized, highly repeatable acupuncture interventions; (4) participants will be instructed to maintain their usual lifestyle for 24 h before scanning, avoiding late nights, smoking, and consumption of coffee or tea. During scanning, participants will close their eyes and wear earplugs. Following the scan, participants will document sensations experienced during the procedure, including somatic sensations, psychological state, and thought activity, to explore the influence of various factors on test outcomes.

In summary, this trial will evaluate the efficacy of acupuncture for PSVV and explore its central mechanisms. It will provide evidence for acupuncture’s effectiveness in treating PSVV and preliminarily investigate its neuroplasticity effects. Additionally, quality control methods will be implemented to enhance the reliability of results. We anticipate this study will offer guidance for the early treatment of PSVV. Findings will be reported according to the Consolidated Standards of Reporting Trials (STRICTA) guidelines (34).
