# Supplementary material for: Effects and central mechanisms of acupuncture for post-stroke vascular vertigo: study protocol of a multicenter, randomized, sham-controlled trial
Source: Front Neurol. 2026 Mar 25;17:1729679. doi: 10.3389/fneur.2026.1729679 (PMC13056849; doi:10.3389/fneur.2026.1729679)
Supplement: Supplementary file 1 [file Supplementary_file_1.pdf]

成都中医药大学附属医院医学伦理委员会

伦理审查批件

**伦理批件号**	2025KL-062		
**项目名称**	针刺治疗卒中后血管性眩晕的临床疗效评价及中枢机制研究		
**项目来源/申办者**	四川省保健科研重点项目（川健研 ZH2025-501）		
**临床研究机构**	成都中医药大学附属医院, 重庆市中医院, 成都市双流区中医医院, 成都市龙泉驿区中医医院		
**本中心主要研究者**	晋松		
**审查类别**	复审审查	**审查方式**	快速审查
**审查日期**	2025-04-15	**审查地点**	成都中医药大学附属医院医学伦理委员会
**审查委员**	马喜桃, 周建伟		
**审查批准文件**	1. 复审申请<br>2. 临床研究方案(20250408, V2.0)<br>3. 知情同意书(20250331, V1.0)<br>4. 招募受试者的材料(20250307, V1.0)<br>5. 病例报告表(20250408, V2.0)		
**审查意见**			

根据国家卫生健康委、教育部、科技部、国家中医药管理局《涉及人的生命科学和医学研究伦理审查办法（2023）》、科技部会同教育部、工业和信息化部、国家卫生健康委等十部委《科技伦理审查办法（试行）（2023）》、国家药监局和国家卫健委《药物临床试验质量管理规范（2020）》、《医疗器械临床试验质量管理规范（2022）》、国家卫生健康委《涉及人的生物医学研究伦理审查办法（2016）》、WMA《赫尔辛基宣言(2016)》和CIOMS《人体生物医学研究国际道德指南（2023）》等伦理原则，经本伦理委员会审查，同意在本机构按所批准的文件开展本临床研究。

1.请遵循GCP原则、按照伦理委员会批准的方案尽快启动临床研究。研究实施前，请按国家医学研究登记备案信息系统要求如实、完整、准确上传资料，并根据研究进展及时更新信息。

2.研究过程中，请按照法规及指南要求提交修正案申请，年度/定期进展报告，安全性报告，违背/偏离方案报告，暂停/终止研究报告，研究完成报告。

发生为消除对受试者紧急危害的研究方案的偏离或者修改；增加受试者风险或者显著影响研究实施的改变；所有可疑且非预期严重不良反应；可能对受试者的安全或者临床研究的实施产生不利影响的新信息，请及时报告伦理委员会。

3.对于涉及人类遗传资源的研究，请严格遵照人类遗传资源管理条例相关规定，及时完成向中国人类遗传资源管理办公室的申报备案工作，获得批准后才能启动研究。

**批件有效期**	2025.04.15-2028.04.14	**跟踪审查频率**	12 个月
**联系人及联系方式**	马喜桃: 028-87783142, ethicscd@126.com		
**主任委员签字**	蒋运兰		

成都中医药大学附属医院医学伦理委员会

（盖章）

日期：2025年4月15日

6100720043
