# Supplementary material for: Effects and central mechanisms of acupuncture for post-stroke vascular vertigo: study protocol of a multicenter, randomized, sham-controlled trial
Source: Front Neurol. 2026 Mar 25;17:1729679. doi: 10.3389/fneur.2026.1729679 (PMC13056849; doi:10.3389/fneur.2026.1729679)
Supplement: Supplementary file 2 [file Supplementary_file_2.pdf]

Informed Consent Form · Consent Signature Page

Clinical Research Project Title: Clinical Efficacy Evaluation and Central Mechanism Study of Acupuncture for Post-Stroke Vascular Vertigo

Collaborating Institutions: Affiliated Hospital of Chengdu University of Traditional Chinese Medicine, Chongqing Hospital of Traditional Chinese Medicine, Shuangliu District Hospital of Traditional Chinese Medicine (Chengdu), Longquanyi District Hospital of Traditional Chinese Medicine (Chengdu)

I have read the above introduction to this study, and have been afforded the opportunity to discuss the study with the doctor and raise questions. All questions I put forward have received satisfactory responses.

I am aware of the potential risks and benefits of participating in this study. I understand that participation is voluntary, confirm that I have had sufficient time to consider this decision, and clearly acknowledge the following:

- I may consult the doctor for additional information at any time;
- I may withdraw from this study at any time without facing discrimination or retaliation, and my medical treatment and rights will not be affected.

I also understand that if I withdraw from the study midway, it will be of great benefit to the overall study if I inform the doctor of any changes in my condition and complete the corresponding physical and chemical examinations.

Should I require any other medication due to changes in my condition, I will either consult the doctor in advance or truthfully inform the doctor afterwards.

I consent to the project undertaking institution, clinical trial research institutions, and ethics committee accessing my research data.

I will receive a signed and dated copy of this Informed Consent Form.

Finally, I decide to consent to participating in this study and commit to adhering to the doctor's advice as much as possible.

Signature of Subject or Family Member: _______________

Date: _______ (Year/Month/Day)

Contact Phone Number: _______________

I confirm that I have explained the details of this study to the subject, including potential benefits and risks, and have provided the subject with a signed copy of this Informed Consent Form.

Doctor’s Signature: _______________

Contact Phone Number: _______________

知情同意书·同意签字页

临床研究项目名称：针刺治疗卒中后血管性眩晕的临床疗效评价及中枢机制研究

协作单位：成都中医药大学附属医院、重庆市中医院、成都市双流区中医医院、成都市龙泉驿区中医医院

我已经阅读了上述有关本研究的介绍，而且有机会就此项研究与医生讨论并提出问题。我提出的所有问题都得到了满意的答复。

我知道参加本研究可能产生的风险和受益。我知晓参加研究是自愿的，我确认已有充足时间对此进行考虑，而且明白：

- 我可以随时向医生咨询更多地信息；
- 我可以随时退出本研究，而不会受到歧视或报复，医疗待遇与权益不会受到影响。

我同样清楚，如果我中途退出研究，我若将我的病情变化告诉医生，完成相应的理化检查，这将对整个研究十分有利。

如果因病情变化我需要采取任何其他的药物治疗，我会事先征求医生的意见，或事后如实告诉医生。

我同意课题承担单位、临床试验的研究单位及伦理委员会查阅我的研究资料。

我将获得一份经过签名并注明日期的知情同意书副本。

最后，我决定同意参加本项研究，并保证尽量遵从医嘱。

受试者或家属签名：______________　　　　　_______年______月______日

联系手机号：______________

我确认已向受试者解释了本研究的详细情况，包括可能的获益和风险。并给其一份签署过的知情同意书副本。

医生签名：______________　　　　　______年______月______日

联系手机号：______________
