# Supplementary material for: Effects and central mechanisms of acupuncture for post-stroke vascular vertigo: study protocol of a multicenter, randomized, sham-controlled trial
Source: Front Neurol. 2026 Mar 25;17:1729679. doi: 10.3389/fneur.2026.1729679 (PMC13056849; doi:10.3389/fneur.2026.1729679)
Supplement: Supplementary file 3 [file Supplementary_file_3.pdf]

Dizziness Handicap Inventory

Items (Symptom Manifestations)	A. Yes	B. No	C. Sometimes
P1 Does looking upward toward the ceiling (with the head in an upward position) exacerbate vertigo?			
E2 Does vertigo or balance disturbance make you feel emotionally low?			
F3 Does vertigo or balance disturbance disrupt your daily work, rest, or travel?			
P4 Does walking between tall goods shelves exacerbate this condition?			
F5 Does vertigo make it difficult for you to get up and lie down in bed?			
F6 Does vertigo or balance disturbance restrict your social activities (e.g., dining out, watching movies, etc.)?			
F7 Does vertigo make it difficult for you to read?			
P8 Does vigorous exercise such as running or dancing exacerbate vertigo?			
E9 Does vertigo make you afraid of staying at home alone?			
E10 Does vertigo make you feel nervous and flustered in front of others?			
P11 Does rapid head turning, tilting the head back, or lowering the head exacerbate vertigo?			
F12 Does vertigo make you afraid of looking down from heights?			
P13 Does the process of turning over while lying flat suddenly exacerbate vertigo?			
F14 Does vertigo restrict you from doing heavy housework or physical labor?			
E15 Does vertigo make you worried that others might mistake you for being drunk?			
F16 Does vertigo prevent you from completing work independently?			
P17 Does walking on zebra crossings exacerbate vertigo?			
E18 Does vertigo make it difficult for you to concentrate?			
F19 Does vertigo prevent you from walking alone in the house at night?			

E20 Does vertigo make you feel scared when staying at home alone?			
E21 Does vertigo make you feel that you have a disability?			
E22 Does vertigo put pressure on your family relationships?			
E23 Does vertigo make you feel frustrated?			
F24 Does vertigo affect your family life or daily work?			
P25 Does bending over exacerbate vertigo?			
**Total Score: DHI-P（ ）; DHI-E（ ）; DHI-F（ ）**			

眩晕障碍量表

症状表现<br>项目	A．是	B．否	C.有时
P1 头朝上仰视眼睛看一下天花板的位置是否会加重眩晕？			
E2 眩晕或平衡障碍是否会让您感到情绪低落？			
F3 眩晕或平衡障碍是否会扰乱您的日常工作或休憩旅行？			
P4 行走于高高的货栏中间是否会加重该疾病？			
F5 眩晕是否会让您感到起床和卧床困难？			
F6 眩晕或平衡障碍是否会使您的社交活动（比如：外出就餐，看电影等）受到限制？			
F7 眩晕是否会使您阅读困难？			
P8 跑步、跳舞等剧烈运动是否会加剧眩晕？			
E9 眩晕是否会使您害怕自己一人独自在家？			
E10 眩晕是否会使您在他人面前感到紧张和慌乱？			
P11 快速的转头、仰头、低头是否会加重眩晕？			
F12 眩晕是否会使您害怕从高处往下看？			
P13 平躺翻身这个过程是否会突然加重眩晕？			
F14 眩晕是否会限制您做较重的家务或进行体力劳动？			
E15 眩晕是否会让您担心害怕他人误认为您喝醉酒了？			
F16 眩晕是否会让您无法单独完成工作？			
P17 行走于斑马线上是否会加重眩晕？			
E18 眩晕是否会让您觉得很难集中注意力？			
F19 眩晕是否会使您夜晚在屋子里活动都不能独自行走？			

E20 眩晕是否会让您独自在家时感到害怕？			
E21 眩晕是否会让您感觉自己患有残疾？			
E22 眩晕是否会给您的家庭关系带来压力？			
E23 眩晕是否会使您产生挫败感？			
F24 眩晕是否会影响您的家庭生活或日常工作？			
P25 弯腰是否会加重眩晕？			
总分 DHI-P（ ）；DHI-E（ ）；DHI-F（ ）			
