# Supplementary material for: Effects and central mechanisms of acupuncture for post-stroke vascular vertigo: study protocol of a multicenter, randomized, sham-controlled trial
Source: Front Neurol. 2026 Mar 25;17:1729679. doi: 10.3389/fneur.2026.1729679 (PMC13056849; doi:10.3389/fneur.2026.1729679)
Supplement: Supplementary file 4 [file Supplementary_file_4.pdf]

Dizziness and Anxiety Rating Scale

<table>
<tr><td rowspan="2">Items</td><td>Scores</td></tr>
<tr><td>0＝No symptoms; 1＝Very mild; 2 ＝Mild; 3 ＝Mild to moderate; 4 ＝Moderate; 5 ＝ Moderate to severe; 6 ＝Severe</td></tr>
<tr><td colspan="2">1.Balance disturbance when standing:
2. Balance disturbance when walking:
3. Current vertigo:
4. Feeling of confusion or disorientation:
5. Overall impression of the condition (from the doctor’s perspective):
6. Overall impression of the condition (from the patient’s perspective):
Total Score:</td></tr>
</table>

眩晕评定量表的评分系统

<table>
<tr><td rowspan="2">目录</td><td>评分</td></tr>
<tr><td>0=无症状；1=很轻；2=轻度；3=轻到中度；4=中度；5=中到重度；6=重度</td></tr>
<tr><td colspan="2">1、站立时平衡失调：
2、行走时平衡失调：
3、现在有眩晕：
4、感到困惑或定向障碍：
5、病情的总体印象（医生角度）：
6、病情的总体印象（患者角度）：
总 分：</td></tr>
</table>
