# Supplementary material for: Effects and central mechanisms of acupuncture for post-stroke vascular vertigo: study protocol of a multicenter, randomized, sham-controlled trial
Source: Front Neurol. 2026 Mar 25;17:1729679. doi: 10.3389/fneur.2026.1729679 (PMC13056849; doi:10.3389/fneur.2026.1729679)
Supplement: Supplementary file 5 [file Supplementary_file_5.pdf]

Vertigo Diary

**Item/No.**	**1**	**2**	**3**	**4**
Vertigo Onset Time				
Vertigo Offset Time				
VAS Score (1-10)<br>(See Instruction 1)				
Vertigo Severity Score (0-3)<br>(See Instruction 2)				
Whether Relevant Medications Are Taken<br>(If yes, please fill in the medication name, dosage, administration time, relief time, drug side effects, etc.)				

Instruction 1

VAS Score: Please indicate the position corresponding to the severity of your vertigo.

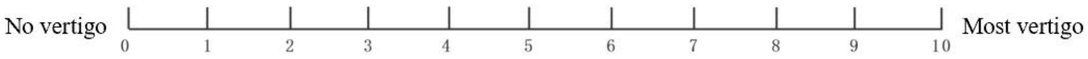

Instruction 2

Vertigo Severity Score:

- 0 = No vertigo
- 1 (Mild) = Vertigo occurs but does not affect daily activities
- 2 (Moderate) = Vertigo occurs and affects daily activities
- 3 (Severe) = Unable to perform daily activities due to vertigo

Instruction 3

Time should be filled in 24-hour format as follows: Year ____ Month ____ Day ____ Hour ____ Minute ____

Instruction 4

For the record of medication administration, please fill in:

Medication Name: ________; Dosage: ____ tablets per dose, ____ doses per day; Total administration duration: ____ days.

眩晕日记

项目编号	1	2	3	4
眩晕开始时间				
眩晕结束时间				
VAS 评分 1-10<br>（参看说明 1）				
眩晕程度评分 0-3<br>（参看说明 2）				
是否服用相关药物<br>（如有请填写药物名称、剂量、使用时间、缓解时间、药物副作用等）				

说明 1：

VAS 评分：请读出您眩晕轻重的位置

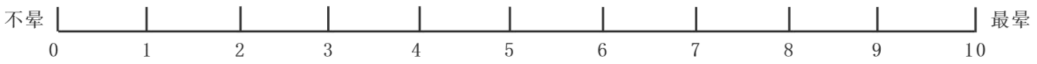

说明 2：

眩晕程度评分：

0=没有眩晕

1（轻度）=眩晕但不影响日常活动

2（中度）=眩晕但影响日常活动

3（重度）=因眩晕不能进行日常活动

说明 3：

时间填写格式按 24 小时制　　年　月　日　时　分

说明 4：

药物服用情况记录需填写　药物名称　片/次，　次/天，共服用　天。
