# Supplementary material for: Effects and central mechanisms of acupuncture for post-stroke vascular vertigo: study protocol of a multicenter, randomized, sham-controlled trial
Source: Front Neurol. 2026 Mar 25;17:1729679. doi: 10.3389/fneur.2026.1729679 (PMC13056849; doi:10.3389/fneur.2026.1729679)
Supplement: Supplementary file 6 [file Supplementary_file_6.pdf]

36-Item Short Form Health Survey

**1. In general, how would you rate your overall health? (Please tick the corresponding number)**	1 Excellent; 2 Very good; 3 Good; 4 Fair; 5 Poor
**2. Compared with one year ago, how would you rate your overall health now?**	1 Much better; 2 Somewhat better; 3 About the same; 4 Somewhat worse; 5 Much worse
**3. The following questions are about your daily activities. Does your health limit these activities? If yes, to what extent?<br>1 Very limited; 2 Somewhat limited; 3 Not limited**	
(1) Vigorous physical activities (e.g., running, lifting heavy objects, participating in intense exercises)	1 2 3
(2) Moderate activities (e.g., moving tables and chairs, mopping the floor, doing calisthenics)	1 2 3
(3) Lifting or carrying groceries (e.g., going shopping for daily food)	1 2 3
(4) Climbing several flights of stairs	1 2 3
(5) Climbing one flight of stairs	1 2 3
(6) Bending, kneeling, or stooping	1 2 3
(7) Walking about 1,500 meters	1 2 3
(8) Walking about 800 meters	1 2 3
(9) Walking about 100 meters	1 2 3
(10) Taking a shower and getting dressed by yourself	1 2 3
**4. During the past month, have your work and daily activities been affected by your physical health in the following ways?**	
(1) Reduced the time spent on work and other activities	1 Yes; 2 No
(2) Only completed part of the things you planned to do	1 Yes; 2 No
(3) Limited the types of work or activities you could do	1 Yes; 2 No
(4) Had difficulty completing work and activities (e.g., needed extra effort)	1 Yes; 2 No
**5. During the past month, have your work and daily activities been affected by emotional problems (e.g., depression or anxiety) in the following ways?**	
(1) Reduced the time spent on work and other activities	1 Yes; 2 No
(2) Only completed part of the things you planned to do	1 Yes; 2 No
(3) Could not concentrate on work and other activities as usual	1 Yes; 2 No
**6. During the past month, to what extent have your physical health and emotional problems affected your daily social activities with family, friends, neighbors, colleagues, or community groups?**	1 No effect at all; 2 Slight effect; 3 Moderate effect; 4 Large effect; 5 Extreme effect
**7. During the past month, how severe was the physical pain you experienced?**	1 No pain; 2 Very mild pain; 3 Mild pain; 4 Moderate pain; 5

	Severe pain; 6 Very severe pain
**8. During the past month, to what extent has physical pain affected your normal work (including outdoor work and housework)?**	1 No effect; 2 Slight effect; 3 Moderate effect; 4 Large effect; 5 Extreme effect
**9. The following questions are about your feelings and situation during the past month. Please choose the answer that best matches your experience. How often did you feel this way?**<br>**1 All the time; 2 Most of the time; 3 A good bit of the time; 4 Some of the time; 5 A little of the time; 6 None of the time**	
(1) Did you feel that your life was full?	1 2 3 4 5 6
(2) Did you feel tense?	1 2 3 4 5 6
(3) Did you feel downhearted and that nothing could cheer you up?	1 2 3 4 5 6
(4) Did you feel calm and peaceful?	1 2 3 4 5 6
(5) Did you have a lot of energy?	1 2 3 4 5 6
(6) Did you feel down and depressed?	1 2 3 4 5 6
(7) Did you feel worn out?	1 2 3 4 5 6
(8) Did you feel happy?	1 2 3 4 5 6
(9) Did you feel bored?	1 2 3 4 5 6
**10. Have your health or emotional problems limited your social activities?**	1 All the time; 2 Most of the time; 3 A good bit of the time; 4 Some of the time; 5 None of the time
**11. For the following statements, please indicate whether they are true or false for you. Choose the answer that best fits your situation.**<br>1 Definitely true; 2 Mostly true; 3 Unsure; 4 Mostly false; 5 Definitely false	
(1) I seem to get sick more easily than others	1 2 3 4 5
(2) I think my health is as good as that of others	1 2 3 4 5
(3) I think my health is getting worse	1 2 3 4 5
(4) My health is very good	1 2 3 4 5

Scoring Formula: Converted Score = (Actual Score - Minimum Possible Score for the Domain) ÷ (Maximum Possible Score for the Domain - Minimum Possible Score for the Domain) × 100

生活质量评估量表

**1、您认为您的健康状况总体上来说属于<br>（请在相应数字上打勾）**	1 极好　2 非常好　3 好　4 一般　5 差
**2、与一年前相比，您认为您现在的健康状况总体上来说？**	1 好很多 2 好一点　3 一样 4 差一点　5 差很多
**3、下面是关于您的日常活动相关的问题，您的健康状况是否限制您的这些活动？如果有限制，程度如何？<br>1 限制很多，　2 限制一点，3 没有限制**	
（1）重体力活动（如跑步、搬重物、参加一些较为剧烈的活动）	1　2　3
（2）适度活动（移动桌椅、清扫地板、做操）	1　2　3
（3）手提日常食品（如上街买菜、购物等）	1　2　3
（4）上几层楼梯	1　2　3
（5）上一层楼梯	1　2　3
（6）能够弯腰、屈膝、俯身	1　2　3
（7）步行 1500 米左右路程	1　2　3
（8）步行 800 米左右路程	1　2　3
（9）步行 100 米路程	1　2　3
（10）自己冲凉和穿衣	1　2　3
**4、在最近一个月内，您的工作和日常活动中，是否由于您的身体健康原因而带来了下列问题？**	
（1）缩减了工作量和其它活动的时间	1 有　2 没有
（2）本来想要做的事情只能完成一部分	1 有　2 没有
（3）想要做的工作或活动的种类受到限制	1 有　2 没有
（4）难以完成这些工作和活动（如：需额外努力）	1 有　2 没有
**5、在最近一个月内，您的工作和日常活动有没有因为您的心情、情感问题（如：消沉或忧虑）引发下列问题而出现以下情况？**	
（1）缩减了工作和其它活动的时间	1 有　2 没有
（2）本来想要做的事情只能完成一部分	1 有　2 没有
（3）您不能像通常一样专心地做这些工作和其它活动	1 有　2 没有
**6、在最近一个月内，您的身体健康和情绪不好在多大程度上影响了您与您的家人、朋友、邻居、同事或集体之间的日常社交活动？**	1 根本没影响<br>2 稍微有点影响<br>3 中等程度影响<br>4 较大影响<br>5 极大影响
**7、在最近一个月内，您经受过的躯体疼痛有多严重？**	1 无疼痛<br>2 非常轻微疼痛<br>3 有一点疼痛<br>4 中度疼痛<br>5 重度疼痛<br>6 非常严重疼痛

**8、在最近一个月内，身体疼痛多大的程度上影响了您的正常工作（包括户外工作和家务活）？**	1 没影响<br>2 稍微有点影响<br>3 中等程度影响<br>4 较大影响<br>5 极大影响
**9、下列的问题是关于在最近一个月内您的感受如何以及您的情况如何。请给出一个您觉得较接近的答案，您有多少时间有这种感觉？1 所有时间，2 绝大多数时间，3 比较多时间，4 部分时间， 5 偶尔有时， 6 无。**	
（1）您觉得生活充实吗？	1 2 3 4 5 6
（2）您是一个精神紧张的人吗？	1 2 3 4 5 6
(3)您感到垂头丧气,没有什么事能使你欢欣和振作起来？	1 2 3 4 5 6
（4）您是否感到心境平静和安宁？	1 2 3 4 5 6
（5）您是否精力充沛？	1 2 3 4 5 6
（6）您是否感到消沉和忧郁？	1 2 3 4 5 6
（7）您是否感到筋疲力尽？	1 2 3 4 5 6
（8）您是一个快乐的人吗？	1 2 3 4 5 6
（9）您是否已感到厌倦？	1 2 3 4 5 6
**10、您的健康或感情问题限制了您的社交活动吗？**	1 所有时间<br>2 绝大多数时间<br>3 比较多时间<br>4 部分时间<br>5 无
**11、下列这些阐述，对您而言是对还是错？请选出最符合您情况的答案。<br>1 绝对正确，2 大部分正确，3 不能肯定，4 基本错误，5 绝对错误。**	
（1）我好像比别人更容易患病	1 2 3 4 5
（2）我认为我与其他人一样健康	1 2 3 4 5
⑶我认为我的健康状态在变差	1 2 3 4 5
⑷我的健康状况非常好	1 2 3 4 5

换算得分＝（实际得分－该方面的可能最低分）÷（该方面的可能最高分—可能最低分）100
