# Supplementary material for: Effects and central mechanisms of acupuncture for post-stroke vascular vertigo: study protocol of a multicenter, randomized, sham-controlled trial
Source: Front Neurol. 2026 Mar 25;17:1729679. doi: 10.3389/fneur.2026.1729679 (PMC13056849; doi:10.3389/fneur.2026.1729679)
Supplement: Supplementary file 7 [file Supplementary_file_7.pdf]

Treatment Expectation Value

**How effective do you think the treatment for vascular vertigo in this study is?**	Evaluation
**Highly effective** (Symptom improvement ≥ 75%)	☐
**Moderately effective** (Symptom improvement 50%—75%)	☐
**Minimally effective** (Symptom improvement 25%—50%)	☐
**No change** (Symptom improvement 0%—25%)	☐

Notes:

1. The "Highly effective" option is defined as **high expectation**, while all other options are defined as **low expectation**.

2. To eliminate confounding bias, this assessment was conducted by a physician who was **blinded to the group allocation** of participants.

治疗期待值

**受试者认为本课题治疗血管性眩晕的疗效如何？**	评价
疗效很好（症状改善 75％以上）	☐
疗效一般（症状改善 50％—75％）	☐
有一点疗效（症状改善 25％—50％）	☐
没有变化（症状改善 0％—25％）	☐

注：

1.“疗效很好”选项为高期待，其余选项为低期待；

2. 为排除干扰因素，此项由一名不知道具体分组情况的医师进行评估。
